# Bacteriophage replication strategies are associated with organic matter energy content on coral reefs

**DOI:** 10.1128/msystems.00395-26

**Published:** 2026-06-22

**Authors:** Natascha S. Varona, Lisa Schellenberg, Will Barnes, Yun Scholten, Andreas F. Haas, Cynthia Silveira

**Affiliations:** 1 Department of Biology, University of Miami 5452, Coral Gables, Florida, USA; 2 Department of Environmental Science, Policy, & Management, University of California Berkeley 1438, Berkeley, California, USA; 3 NIOZ Royal Netherlands Institute for Sea Research, Texel, the Netherlands; 4 Institute for Biodiversity and Ecosystem Dynamics, University of Amsterdam, Amsterdam, the Netherlands; 5 GELIFES, University of Groningen, Groningen, the Netherlands; 6 Rosenstiel School for Marine, Atmospheric and Earth Sciences, University of Miami 5452, Miami, Florida, USA; University of Florida, Gainesville, USA; Universidade Federal do Rio de Janeiro, Rio de Janeiro, Brazil

**Keywords:** bacteria, virus, exometabolome, dissolved organic matter

## Abstract

Bacteriophages, viruses that infect bacteria, play a crucial role in carbon cycling within marine environments. In coral reefs, dissolved organic matter (DOM) released by benthic primary producers such as algae fuels heterotrophic microbial growth, which can be detrimental to corals. This microbialization process has been associated with the abundance and replication strategies of bacteriophages, but the direct relationship between reef DOM composition and bacteriophage communities remains unclear. Here, we combine metabolomics, metagenomes, and viromes to demonstrate that phage communities have significant relationships with DOM composition on the reefs of Curaçao, Southern Caribbean. While total viral abundances did not significantly correlate with overall dissolved organic carbon (DOC) concentration on these reefs, co-occurrence networks identified thousands of statistically significant associations between free or cell-associated viruses and organic compounds. Cell-associated phages had significantly more positive associations with compounds that had a reduced nominal oxidative state of carbon (NOSC). Furthermore, temperate phages were more frequently correlated with metabolites exhibiting higher Gibbs energy than putatively lytic phages. Six of the ten viruses with the highest number of positive associations with metabolites were temperate (i.e., encoded an integrase or were identified as a prophage), despite this network consisting of approximately 90% lytic viruses. These temperate viruses were predicted to infect members of the genus *Sphingobium*. Together, these findings reveal a connection between phage replication strategies and DOM energy availability, with potential implications for coral reef biogeochemistry.

**IMPORTANCE:**

Coral reefs are highly dynamic ecosystems where microbial communities and organic matter cycles are intricately linked. This study provides new insights into how bacteriophages interact with dissolved organic matter (DOM) composition, revealing that cell-associated bacteriophages, particularly temperate phages, are associated with more energy-rich organic compounds. These findings suggest that DOM could affect the lysis-lysogeny decision of temperate phages or that lysogeny may play an underappreciated role in shaping the reef carbon cycle. Energy-rich organic compounds have generally been associated with increased algal abundances and coral decline. By demonstrating significant connections between viral infection strategies and the energy content of DOM, our results highlight the potential for phages to influence coral reef biogeochemistry and health.

## INTRODUCTION

Viruses play crucial roles in marine ecosystems by driving carbon cycling through cell lysis (1–4). It is estimated that every second, there are approximately 10^22^ viral infections occurring in the ocean (2). Bacteriophage-driven lysis of microorganisms releases organic matter and nutrients into the environment, diverting energy from the secondary consumers of the food web (e.g., zooplankton), toward predominantly heterotrophic bacteria, a process known as the viral shunt (3). Additionally, lysed organic matter can aggregate with cell debris and mucus, forming marine snow and accelerating carbon export to the ocean floor (5). Viral lysis is estimated to release approximately 145 Gt of carbon per year in the tropical and subtropical oceans (1).

In coral reef ecosystems, carbon fluxes have been shown to influence reef health (6–8). The Disease, Dissolved organic matter, Algae, and Microorganisms (DDAM) model (9, 10) describes a positive feedback loop in which increased dissolved organic matter (DOM) released by fleshy algae fuels heterotrophic microbial growth, leading to oxygen depletion that can cause coral death. The newly opened niche space is taken over by algae, which can now perpetuate the DDAM loop by releasing more DOM. Among reef organisms, algae exhibit the highest rates of DOM exudation, with conservative estimates suggesting that turf and macroalgae release approximately 10% of their photosynthetically fixed organic carbon (11, 12). While both corals and algae contribute to the majority of reef DOM, algae exude more labile DOM and reduced organic molecules compared to corals (13, 14). These compounds are generally more energy-rich and provide a greater metabolic potential per carbon (12, 15, 16), which affects the microbial community composition in reef waters (13, 14, 17, 18). DOM from macroalgae promotes decreased microbial diversity and the growth of copiotrophic taxa such as *Gammaproteobacteria* (13). In contrast, DOM from corals is associated with high microbial diversity and an enrichment of *Alphaproteobacteria* (13). A study that analyzed over 400 samples from 60 coral reef sites revealed that the microbial consumption of algal-derived DOM leads to an increase in microbial biomass and a shift in community composition, a phenomenon described as coral reef microbialization (19). The algae-derived exudates are more energy-rich, reducing bacterial growth efficiency to as low as 6%, whereas coral exudates can be metabolized more efficiently at 18% (13). The low-efficiency consumption of algae compounds increases biological oxygen demand and contributes to microbially driven deoxygenation (11, 19, 20). These combined effects further shift reef community dynamics in favor of fleshy and turf algae (9, 10).

Viruses may be an integral part of the DDAM pathway (21). They may do so by directly lysing different bacterial members of the community (7, 22–24) and releasing DOM into the environment via cell lysis (5, 6), which, in turn, selects for different microbes, or by switching replication strategies (lysis and lysogeny) that may select for a distinct bacterial community composition. Healthy coral reefs are typically associated with high viral predation pressure (7), with phages frequently targeting *Gammaproteobacteria* (23). However, as bacterial abundance increases, viral replication strategy shifts toward lysogeny, where the viral genome integrates into the host chromosome rather than lysing the cell (22). Through lysogeny, viruses can influence bacterial gene expression, potentially altering microbial functions. For instance, phages in coral reefs have been found to encode genes related to energy, amino acid, and carbohydrate metabolism, as well as the biosynthesis of secondary metabolites, all of which can impact host metabolism (25). Viral reprogramming can modify key metabolic pathways, such as the pentose phosphate pathway, tricarboxylic acid cycle, and glycolysis (26, 27). Additionally, phages can introduce virulence genes (28, 29) and antibiotic resistance genes (30), further influencing and altering microbial dynamics.

While the pairwise relationships between phages and bacteria and between bacteria and DOM have been described in coral reefs, the direct relationship between phages and DOM remains largely unexplored. Understanding this relationship is of particular importance given that DOM sources could have bottom-up influences on viral infection through changes in host metabolism, or that viruses can top-down control DOM release through viral lysis or manipulation of host metabolism, affecting DOM composition in the environment. To investigate the relationship between phages and DOM composition on coral reefs, this study integrates total dissolved organic carbon (DOC) and nitrogen concentrations, microscopy-derived cell and viral abundances, size-fractionated metagenomics and viromics, and exometabolomic data from coral reefs in Curaçao. To determine the energy availability of organic compounds, we calculated the nominal oxidation state of carbon (NOSC) per compound, which can be used as a proxy to establish bioenergetic potential for catabolism (31). By constructing a co-occurrence network between organic compounds and phage abundances derived from metagenomes and viromes, we identified thousands of significant associations, revealing a relationship between temperate viruses and energy-rich compounds.

## MATERIALS AND METHODS

### Sampling

**Fig 1:**
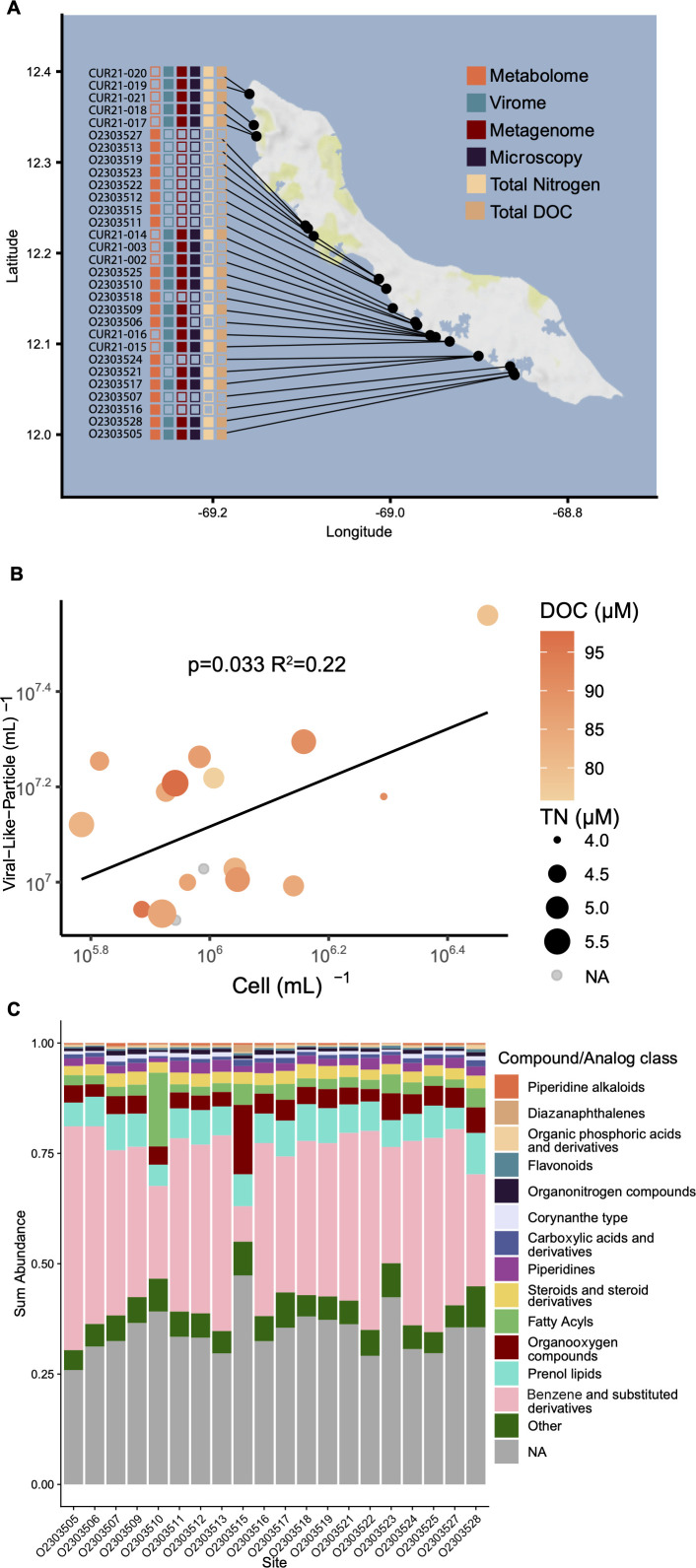
Relationships between viral and microbial abundance and organic matter content. (**A**) Map of Curaçao showing the 29 sampling sites where exometabolomes, viromes, metagenomes, microscopy, total nitrogen, and total DOC were collected. (**B**) Linear regression between virus-like-particle abundances and cell abundances, showing a significant positive relationship based on log_10_-normalized data, *P*-value = 0.033, *R*^2^ = 0.22. The color intensity indicates DOC concentration, and the size represents the total nitrogen concentration, which showed no significant correlations with virus-like-particle abundances, cell abundances, or virus-to-microbe ratios. (**C**) Relative abundance of classified features and analogs at the class level in the exometabolomes by site.

Exometabolomes (*n* = 20), total DOC (*n* = 15), total nitrogen (TN; *n* = 15), viromes (*n* = 17), metagenomes (*n* = 18), and microscopy data (*n* = 16) were sampled on 29 distinct sites on the leeward side of Curaçao in the Caribbean (Fig. 1A). Viromes, metagenomes, and microscopy data were previously published in Varona et al. (23. Total DOC, nitrogen, and exometabolomes are described here for the first time. The coral reef benthic boundary layer (<30 cm above the reef benthos) (32) was sampled using SCUBA at a depth between 5 and 10 m in July–October 2021 (Table S1). Briefly, seawater was collected via a sample-washed bilge pump into 5-gallon collapsible carboys, collecting sufficient volume to avoid potential problems with losses and filtration steps in preparation of subsampling for metagenomes, microscopy, and viromes (23). Custom-made Niskin bottles (2 L) were used to collect samples for DOC, nitrogen, and exometabolomes. Each 2 L sample was subdivided into smaller samples consisting of the following final volumes: 40 mL for DOC, 4 mL for nutrients, and 1 L for metabolomes. Sample containers were rinsed with sample water before collection. All samples were transported to the molecular laboratory of the Caribbean Marine Biological Institute (CARMABI) and processed within 2 h of collection. Seven sites have all data types (metagenome, virome, microscopy, DOC, nitrogen, and exometabolome), while the other sites have gaps in the data availability (Table S1).

For metagenomes and viromes, upon arrival in the laboratory, 2 L seawater subsamples from the carboys were pre-filtered with 8 µm filters (Whatman, Milwaukee, USA) at an approximate flow rate of 12 mL/min using a peristaltic pump Masterflex L/S (Avantor, Gelsenkirchen, Germany). Cells were collected on 0.22 µm Sterivex filters (Millipore Sigma, Burlington, VT, USA) for metagenome sequencing. These filters were stored at −80°C and kept frozen during transport until DNA extraction with Nucleospin Tissue Kit (Macherey-Nagel, Duren, Germany). For viromes, 5 L subsamples were pre-filtered using 8 µm and 0.45 µm filters and concentrated using a Tangential Flow Filtration system, Vivaflow 100 kDa cassette (Sartorius, Goettingen, Germany) . Samples were treated with chloroform (0.1%) and stored at 4°C until precipitation with polyethylene glycol 8000 (10%) and DNA extraction with the Purelink Viral DNA kit (ThermoFisher, Carlsbad, CA, USA). The remaining water volume from the carboys was discarded.

### Genomic analyses

Sequencing of metagenomes and viromes, viral identification, and abundance calculations were described in Varona et al. (23). Briefly, total DNA for metagenomes and viromes was sequenced on an Illumina HiSeq 4000 (2 × 150 bp). Reads were trimmed and quality filtered with BBDuk v.38 (33), assembled into contigs using metaSPAdes v3.15.5 (34), which, to date, is one of the most efficient and widely used assemblers of viral contigs (34, 35). Six selected samples collected on a 0.22 µm filter underwent MetaHi-C processing with Phase Genomics’ ProxiMeta Hi-C kit (Seattle, WA, USA). Viral genomes were identified using VIBRANT v1.2.1 (36), dereplicated with CD-HIT v4.8.1 (37), binned with vRhyme v1.1.0 (38), and required a minimum length of 10,000 kb or, if smaller, were assessed for high or complete quality with CheckV v1.0.1 (39). Contigs containing contamination, as determined by CheckV, were removed for gene analyses. vMAGs were clustered based on 80% gene content and 95% nucleotide identity using Virathon v.1 (40), and viral abundances were estimated using genome length-adjusted read counts. The lengths and abundances of viral genomes and gene fragments are publicly available through Figshare (https://doi.org/10.6084/m9.figshare.28924508.v2). Viral host predictions for the viruses with the highest number of statistically significant correlations were made using iPHoP v1.3.3-0 with the most recent available database (August 2023), and only matches with iPHoP scores greater than 0.9 are reported (41). Few viruses had existing host information from a previous proximity-ligation study, including *Sphingobium yanoikuyae* (23). Viral replication strategies were predicted through VIBRANT v1.2.1, which identifies temperate viruses, that is, those with the ability to establish lysogenic infections, based on the presence of integration genes or if viral sequences are observed inside a bacterial contig. All other viruses are classified as “putatively lytic,” which may be an inflated category that contains false positives (i.e., unrecognized temperate viruses). Here, we apply the temperate and lytic terminology throughout the text following recommendations in the field (42).

Prokaryotic MAGs were binned from contigs resulting from SPADES v3.15.5 and MEGAHIT v1.2.9 co-assembly (43) of the 0.22 µm fraction using CONCOCT v1.0 (44) and MetaBAT2 v2.12.1 (45). In addition, for proximity-ligated samples, prokaryotic MAGs were assembled using the cross-linking information through Proximeta (46). Bins were refined with MetaWRAP (47) and their quality assessed using CheckM (48). To capture Hi-C phage–host linkages, bMAGs with >20% completion and <10% redundancy were retained, then dereplicated with fastANI at 95% identity and classified using GTDB-tk v2 (49).

### Dissolved organic matter processing and extraction

The water samples for exometabolomics were processed using solid-phase extraction of DOM (SPE-DOM) with modifications (50, 51). Each sample was filtered through a 0.22 μm Sterivex-GP filter (Millipore, Burlington, Massachusetts, USA) at ~1 L/h. After discarding the initial 200 mL, 1.1 L of filtrate was collected, acidified to pH 2 with 36% ultrapure HCl, and extracted using a 200 mg Bond Elut-PPL resin cartridge (3 mL; Agilent 12105005). Before the field campaign, PPL cartridges were preconditioned with 3 mL of LC-MS grade MeOH (≥99.9% MeOH; Honeywell, Riedel-de Haën), soaked overnight in MeOH, rinsed twice with de-ionized water, and dried after a final MeOH rinse. Immediately prior to sample processing, PPL cartridges were activated with 3 mL of MeOH and two fills of pH 2 water. Samples were extracted at ~5 mL/min using a Masterflex L/S (Avantor, Gelsenkirchen, Germany), and acid-leached tubing (0.1 M HCl), as well as flushed with 100–200 mL of sample. Cartridges were desalinated with three fills of pH 2 water and dried with nitrogen gas. The cartridges were stored at −80°C. Prior to liquid chromatography coupled with tandem mass spectrometry (LC-MS/MS) analysis, DOM was eluted with 2 mL LC-MS grade MeOH into combusted 1.5 mL LC glass vials (BGB Analytik AG, Boeckten, Switzerland). The extracts were dried overnight in a vacuum centrifuge, re-dissolved in 100 μL LC-MS grade MeOH, and transferred to a combusted glass insert.

### Exometabolomic analysis

An aliquot of 5 µL of DOM extract was injected into an ultra-high-performance liquid chromatography system (UHPLC) (Agilent 1290 Infinity II), equipped with a thermostatted auto-injector and column compartment, coupled to a Q-Exactive Plus Orbitrap Mass Spectrometer (Thermo Fisher Scientific, Bremen, Germany) equipped with an Ion-max source with a HESI (Heated Electrospray Ionization) probe, using positive ionization mode (52). Chromatographic separation was performed using a C18 core-shell column (Kinetex, 150 × 2 mm, 1.8 µm, 100 Å; Phenomenex) at 30°C with a flow rate of 0.5 mL/min. Solvent A was H_2_O + 0.1% formic acid (FA); solvent B was acetonitrile + 0.1% FA. Elution program was as follows: 0.5 min at 5% B, linear gradient to 50% B over 7.5 min, to 99% B over 2 min, 7 min wash at 99% B, and 8 min re-equilibration. Ion source settings were as follows: sheath gas 70 AU, auxiliary gas 15 AU, probe temperature 400°C, spray voltage 3.5 kV, capillary temperature 320°C, and S-Lens 50 V. The maximum ion injection time was 100 ms with automated gain control (AGC) targets of 1 × 10^6^ for MS1 and 3.0 × 10^5^ for MS/MS, with a 10% minimum C-trap AGC threshold. Compounds were detected in the *m*/*z* 150–1,500 range at 70,000 resolution (*m*/*z* 200) and one micro-scan per MS1. MS/MS spectra were acquired in DDA mode for the top five abundant ions, with 17,500 resolution and one micro-scan. Fragmentation used a stepwise collision energy (20%–40%) with *z* = 1. MS/MS experiments were triggered 2–15 s after peak apex, with dynamic exclusion set to 5 s and 3 ppm mass tolerance. Quality controls included a Sulfa mix (0.01 µg/mL; Agilent STD6.1, sulfamethizol, sulfamethazine, sulfachloropyridazine, sulfadimethoxine), and a control seawater sample at the start and end of the sequence, as well as MeOH injections at the start, end, and between every 5 samples.

The data from liquid chromatography-tandem mass spectrometry were processed in MZmine3 (53), followed by spectral matching with “Global Natural Products Social Molecular Networking (GNPS)” (https://gnps.ucsd.edu) (54) using the feature-based molecular networking workflow (FBMN) (55). MS/MS raw datafiles were converted to .mzML in centroid mode using MSConvert (Proteowizard) and consequently processed with MZmine3 (version 3.9.0). Mass detection was performed in centroid mode with a minimum signal-height threshold of 1.0 × 10^5^ for MS^1^ and 1.0 × 10^3^ for MS^2^ levels. Features were generated using the ADAP chromatogram builder (56) based on extracted ion chromatograms (XICs) from each detected *m*/*z* value, requiring a minimum of four consecutive scans. Parameters included a group intensity threshold of 1.0 × 10^5^, a minimum highest intensity of 2.0 × 10^5^, and a scan-to-scan *m*/*z* tolerance of 0.0015 *m*/*z* or 10 ppm. The local minimum feature resolver was used to separate overlapping or co-eluting peaks. MS/MS scan pairing was activated, the precursor tolerance was set to 0.010 *m*/*z* or 10 ppm, and the retention time tolerance was 0.050 min. Chromatographic settings included an 85% threshold, 0.08 min minimum search range, and minimum absolute peak height of 2.0 × 10^5^. Additional settings included a minimum peak top-to-edge ratio of 1.4, a peak duration range of 0.00–2.0 min, and a minimum of four scans. Isotope peak grouping applied a 13C filter with *m*/*z* tolerance of 0.001 or 5 ppm, retention time tolerance of 0.1 min, and a maximum charge of 2, selecting the most intense peak as the monoisotopic ion. XIC alignment across samples was performed using the Join aligner with thresholds of 0.0015 *m*/*z* or 10 ppm (weight = 3), retention time tolerance of 0.150 min (weight = 1), and mobility weight of 1. Join Aligner parameters were selected to prioritize high-confidence feature matching across samples; however, these stringent thresholds may reduce sensitivity to rare or low-abundance metabolites with greater retention time variability or mass inaccuracy, potentially excluding true features near the detection limit. Features with at least two isotope peaks, occurring in at least two samples with MS^2^ scans, were retained. Further filtering utilized the multithreaded peak finder with a 10% intensity tolerance, 0.0015 *m*/*z* or 10 ppm sample-to-sample tolerance, 0.15 min retention time tolerance, and a minimum of three scans. Duplicate peaks were filtered with *m*/*z* tolerance of 0.001 or 5 ppm and RT tolerance of 0.05 min. Correlation grouping (metaCorrelate) was applied with a 0.5 min RT tolerance, 3.0E+04 intensity threshold, ≥60% intensity overlap, exclusion of gap-filled features, and feature shape correlation with a minimum of 5 data points and a minimum of 2 data points on edge using Pearson’s coefficient with a minimum feature shape correlation of 85%. Ion identity networking was performed with *m*/*z* tolerance of 0.0015 *m*/*z* or 5 ppm, maximum charge of 2, and maximum cluster size of 2, considering adducts [M-H]⁻, [M + H]^+^, [M + Na]^+^, [M + NH₄]^+^, and modification [M-H_2_O]. Refinement steps included removing networks without monomers and applying adducts and modifications: [M + H]+; [M + Na]+; [M + K]+; [M + NH4]+; [M + 2H]2+; [M + H + NA]2+; [M + H + NH4]2+; [M-H +2NA]+; [M + Ca H]+; [M + Fe H]+ and [M-H_2_O]; [M-2H_2_O]; [M-3H2O]; [M-4H_2_O]; [M-5H_2_O]. Networks smaller than size 2 were removed, and a link threshold of 4 was applied. Lastly, consensus MS/MS of each feature was exported as .mgf files for molecular networking through GNPS.

A feature-based molecular network was created using workflow version 28.2 on the GNPS platform. The spectral network was built with a 0.01 Da precursor and fragment ion mass tolerance, a minimum cosine score of 0.7, and more than four matched peaks. Only the top 15 edges per node were kept, with a maximum connected component size of 200 and a 500 Da precursor shift limit. Consensus spectra were searched against the GNPS library and in analog mode with variable dereplication (54), using a 200 Da maximum mass difference and reporting the top result. Additionally, NPClassifier (57), a tool that uses deep neural networks trained on public data sets, was used to classify the structure of a natural product at three levels into seven Pathways, 70 Superclasses, and 672 Classes.

Data clean-up and normalization were performed using a statistical pipeline for feature-based molecular networks from non-targeted metabolomics data (55). Blanks were removed using a 0.3 threshold, allowing up to 30% blank contribution. Missing values were imputed by replacing zeros with random values between 0 and the minimum value in the blank-removed feature table, based on relative intensity frequencies. Sample-centric normalization (total ion current [TIC]) scaled feature intensities by the total ion current of each sample.

The freely available software R version 4.4.2 (2024-10-31 ucrt) on the platform x86_64-pc-linux-gnu, running under Ubuntu 22.04.3 LTS, was used to clean the metabolomics data set. Data handling and wrangling were performed using the tidyverse collection of packages (58), which uses dplyr, stringr, tidyr, readr, purr, tibble, and forcats for data manipulation, and includes ggplot2 for visualization (59). For analyses conducted within Jupyter Notebooks using IRKernel, IRdisplay was employed to enhance output rendering (60). The KODAMA package was used to perform normalization procedures based on nonlinear dimensionality reduction methods (61). Data processing was performed on 25 runs, where 20 were samples and 5 were blanks.

GNPS annotated 2,703 features as a known compound based on their MS^2^ spectra. Another 14,078 features were matched to highly similar MS^2^ spectra, referred to as analog hits. These analog hits might provide structural information on unknown compounds and correspond to MSI Level 3, indicating structural similarity to a known compound but not an exact identification, whereas library matches were assigned as putative identifications at MSI level 2 (62). The nominal oxidation state of carbon (NOSC) values were calculated based on the predicted formulas, which were derived from the compounds’ Simplified Molecular Input Line Entry System (SMILES) annotation and converted to a chemical formula using a custom script with the R package rcdk. NOSC and Gibbs energy ($\Delta G_{Cox}^{0}$) were manually calculated in R based on the following previously published equations (15, 31).


(1) $$NOSC=-\frac{-Z+4a+b-3c-2d+5e-2f}{a}+4$$

*a*, *b*, *c*, *d*, *e*, and *f* refer to the stoichiometric numbers of the elements C, H, N, O, P, and S, where *Z* corresponds to the net charge of organic compounds, which here was assumed to be 0 for all molecular formulas.

By comparing NOSC values with standard Gibbs energy for oxidation half-reactions, LaRowe and Van Capellen were able to establish an empirical relationship at 25°C and 1 bar (*R* = 0.94), according to the following equation:


$$\Delta G_{Cox}^{0}=60.3-28.5\times NOSC$$

Exometabolite abundances, GNPS matches, analog predictions, and NOSC values are publicly available through Figshare (https://doi.org/10.6084/m9.figshare.28924043) (63).

### Network analyses

To identify associations between viruses and compounds, Pearson correlation networks were constructed for virome (*n* = 7) and metagenome (*n* = 8) data sets separately using the R 4.2.2 package Hmisc 5.2-4 on the University of Miami’s IBM Pegasus supercomputer. Viral abundances are publicly available through Figshare (https://doi.org/10.6084/m9.figshare.28924508). Q-Q plots showed that the data followed a normal distribution with a slight left skew in the metagenome data (Fig. S1 to S3). Only viruses that were present in at least five sites were included in the analysis. Pearson’s values were corrected using the Benjamini-Hochberg method for multiple comparisons, and only associations with corrected *P*-values < 0.01 and correlation coefficients greater than an absolute value of 0.95 were kept for network analysis. We note that we also generated networks using Spearman rank correlations, which produced a 2.4× higher number of free virus associations and a denser network. We assumed that these numbers were inflated and performed subsequent analyses more conservatively using the Pearson correlation network. The network statistics were analyzed using the default Cytoscape v.3.10.3 Analyze network function as undirected to calculate network statistics such as number of nodes, edges, average neighbors, and network density.

In downstream analyses, compounds were grouped by class (if the class was identified) (*n* = 1,913). If the compound class was unknown, the analog class was used (*n* = 12,961). All downstream statistical analyses were performed in RStudio Version 2022.12.0+353. Venn diagrams between metabolites of different samples were computed using the VennDiagram v.1.7.3 package and the venn.diagram() function (64). The wilcox.test() from the stats v.3.6.2 package (65) function was used to compare NOSC with positive and negative associations and with viral lifestyle distributions. Global Fisher’s exact tests were applied to the relationships between viral lifestyle and compound class using fisher.test() while specific drivers were identified using cell-wise Fisher’s exact tests with FDR correction. These tests were performed using Monte Carlo simulations, with 50,000 replicates.

## RESULTS

### Exometabolomic, viral, and bacterial profiles of coral reefs

Across all 29 sampling sites on the leeward coast of Curaçao, DOC concentrations ranged from 75.8 µM to 86.3 µM and total nitrogen (TN) ranged from 4.0 µM to 5.9 µM (Fig. 1B). Virus-like-particle abundance derived from microscopy exhibited a weak but significant linear relationship with cell abundance (linear regression, *P*-value = 0.033, *R*² =0.22) (Fig. 1B). However, total DOC did not exhibit significant correlations with virus-like-particle abundance (linear regression, *P*-value = 0.33), cell abundance (linear regression, *P*-value = 0.53), or the virus-to-microbe ratio (linear regression, *P*-value = 0.77). Similarly, no significant correlations were observed between total nitrogen and virus-like-particle abundance (linear regression, *P*-value = 0.79), cell abundance (linear regression, *P*-value = 0.34), or the virus-to-microbe ratio (linear regression, *P*-value = 0.42). Likewise, the total abundance of viruses in metagenomes and viromes displayed no significant relationship with DOC (linear regression, *P*-value = 0.87 for metagenomes; linear regression, *P*-value = 0.47 for viromes).

Untargeted metabolomics (*n* = 20) using LC-MS/MS identified 32,854 unique features (publicly available on figshare at https://doi.org/10.6084/m9.figshare.28924043 [63]). GNPS annotations identified 2,703 features as known compounds based on their MS² spectra, with an additional 14,078 features classified as analog hits (highly similar MS² spectra, cosine >0.7, GNPS analog mode), resulting in annotations for 42.8% of detected compounds. When accounting for compound abundances, class-level annotated compounds (either library matches or analog hits) made up approximately 65.4% (SD = 5.0%) of detected compounds across samples (Fig. 1C; Table S2). Among these compounds, benzene and substituted derivatives constituted the largest fraction (mean = 35.9%, SD = 9.8%), followed by prenol lipids (mean = 6.9%, SD = 1.2%), organooxygen compounds (mean = 4.7%, SD = 2.7%), and fatty acyls (mean = 3.3%, SD = 3.2%). Among carbohydrates, we found a high relative abundance of glucosamine (mean = 70.2%, SD = 9.2%) and arabinose (mean = 15.6%, SD = 6.39%), sugars indicative of coral exudates (14) (Fig. S4).

### Viruses associate with energy-rich metabolites

**Fig 2:**
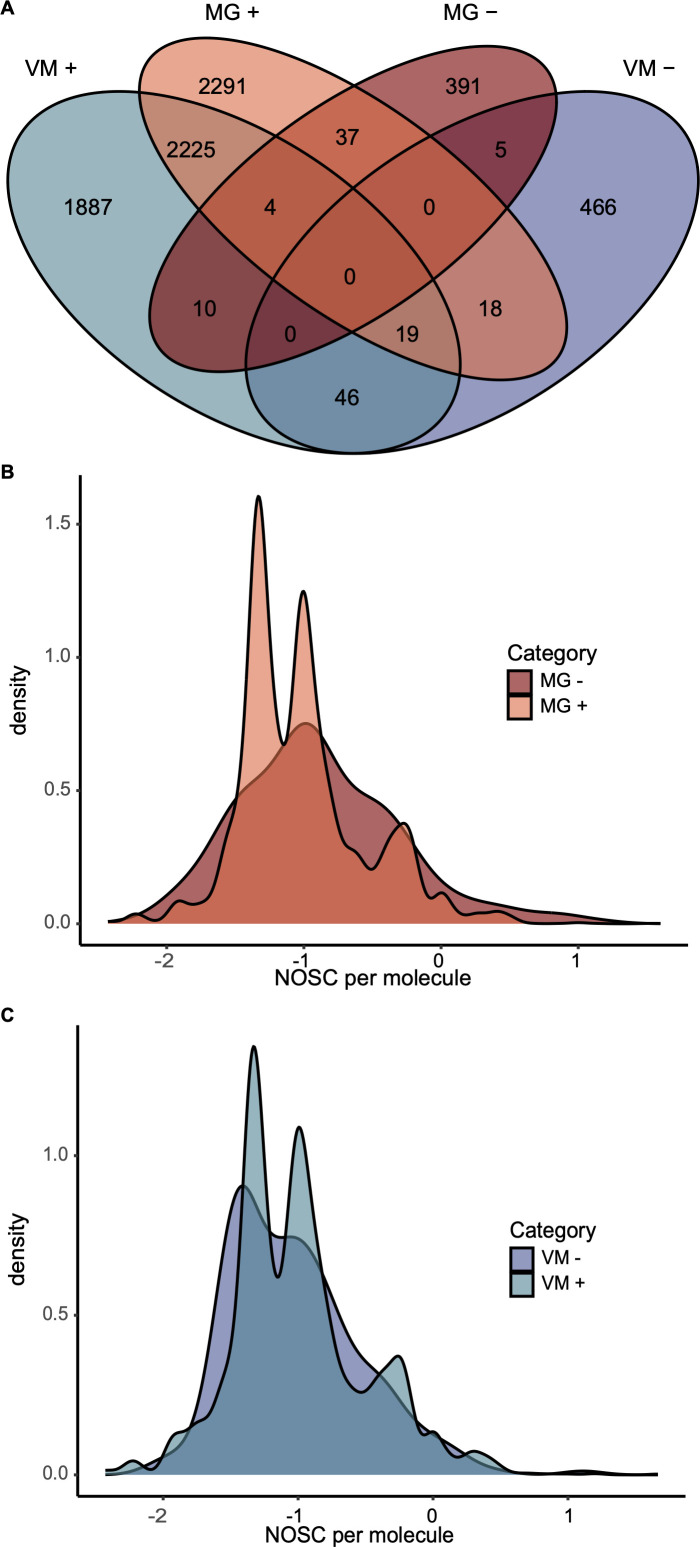
Relationships between viral abundances and metabolites. (**A**) Venn diagram showing the unique metabolites significantly associated (BH-corrected Pearson’s correlation, *P* < 0.01) either positively (+) or negatively (−) with viral abundances from metagenomes (MG) and viromes (VM). (**B**) Density plot comparing the NOSC values for metabolites associated with metagenome-derived viral abundances. Positively associated metabolites exhibited significantly more negative NOSC values (mean = –1.02) compared to negatively associated metabolites (mean = –0.888) (Wilcoxon rank-sum test, *P*-value = 0.000990). (**C**) Density plot comparing NOSC values for metabolites associated with virome-derived viral abundances. No significant difference in NOSC values was observed between positively and negatively associated metabolites (Wilcoxon rank-sum test, *P* = 0.140).

The relationship between viruses (from metagenomes and viromes) and metabolites was further investigated through correlation networks. Pearson correlations yielded 18,619 significant associations (BH-corrected, *P* < 0.01, correlation coefficients > |0.95|) between individual metabolites and metagenome-derived viruses (hereafter referred to as cell-associated viruses) and 15,199 significant associations between individual metabolites and virome-derived viruses (hereafter referred to as free viruses). Most correlations between metabolites and viruses were positive (Fig. 2A). In the cell-associated virus-metabolite network, 4,594 unique metabolites correlated positively, while only 447 were negatively correlated. Similarly, 4,191 unique metabolites were positively associated with free viruses, whereas 554 were negatively associated. Of the positively associated compounds, 2,225 metabolites were shared between metagenome-associated viruses (48.4%) and virome-associated viruses (47.5%). Of the negatively associated compounds, only five metabolites were shared by both free and cell-associated viruses. In all, 134 metabolites appear at the intersection of both positive and negative correlation groups within the same data set (41 have both positive and negative correlations with cell-associated viruses, and 65 have both positive and negative correlations with free viruses), implying that the same metabolite is positively correlated with some viruses and negatively correlated with others.

For metabolites with feature information annotated via GNPS or analog prediction, we calculated the NOSC as a proxy for the potential energy available during catabolism (15). Of the 18,619 associations, 6,431 (34.5%) had NOSC annotations for cell-associated viruses, whereas 5,094 of 15,199 free-virus associations (33.5%) had annotations. Positive correlations between cell-associated viruses and metabolites involved more reduced metabolites (mean NOSC = −1.02) compared to negative correlations (mean NOSC = −0.888, Wilcoxon rank-sum test, *P*-value = 0.000990) (Fig. 2B). Hartigan’s Dip Test revealed a non-unimodal distribution (*P*-value <2.2e−16) of metabolites associated with cell-derived viruses. Gaussian mixture modeling with BIC identified three underlying modes at mean NOSC values –0.3 (*n* = 1,465), −1.2 (*n* = 4,746), and –2.0 (*n* = 157), of which the most pronounced one (–1.2) was further analyzed. By inspecting the increase in density of positive correlations from NOSC values –1.45 to –1.18, these compounds consisted of mainly fatty acyls (71.1%, Fig. S5). Of these fatty acyls, 78.4% were linoleic acids and derivatives. In contrast to the cell-associated virus-metabolite network, no significant differences in NOSC values were observed in the free viral network (Wilcoxon rank-sum test, *P*-value = 0.140), although the lack of differences in the latter may be a result of lower statistical power due to a lower effect size in the free virus network (Fig. 2C).

### Correlations between metagenome-derived viral abundances and metabolites

**Fig 3:**
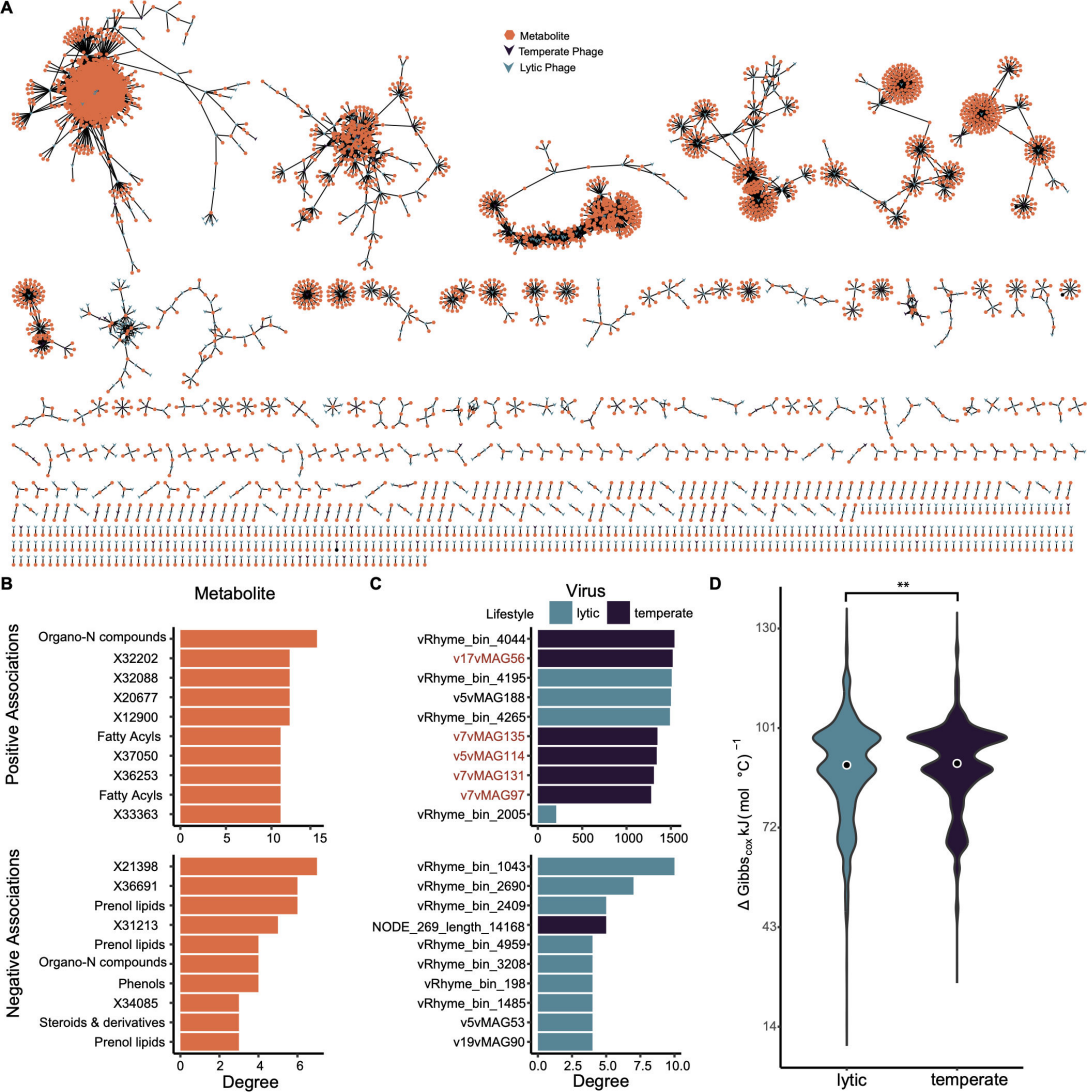
Network of interactions between metagenome-derived phage abundance and metabolites. (**A**) Network of positive associations between metabolites and phage abundances derived from metagenomes (>0.22 mm fraction). V-shaped nodes represent phages and hexagon-shaped nodes represent metabolites. Edges represent significant positive correlations (Benjamini-Hochman corrected Pearson's, *P*-value < 0.01). (**B**) Metabolites with the most connections with positive associations (top) and negative associations (bottom). If the compound was a previously known compound or matched an analog, the class was included as the *y*-axis label. (**C**) Viruses with the highest number of positive associations (top) and negative associations (bottom). Viruses highlighted in red have previously been reported to be associated with the host *Sphingobium yanoikuyae*. (**D**) Gibbs free energy of metabolites associated with putatively lytic and temperate viruses (Wilcoxon rank-sum test, *W* = 4,993,937, *P*-value = 0.00404, lytic mean = 88.8 KJ [mol °C]^−1^; temperate mean = 89.9 KJ [mol °C]^−1^).

Of the 18,619 significant edges in the network connecting metabolites and the metagenome viruses (>0.22 µm fraction), 18,109 were positive associations (Fig. 3A), and 510 were negative (Fig. S6). The positive network consisted of 5,614 nodes, of which 1,020 were viruses and 4,594 were metabolites. The average number of neighbors was 14.75, indicating that viruses are more frequently associated with multiple metabolites, where viruses formed 1 to 1,541 edges. The network was fragmented into 635 connected components, meaning it consisted of multiple sub-networks rather than a single, fully interconnected system. This structural organization suggests that certain groups of viruses and metabolites interact independently from others, though this may also result from stringent cutoffs for the detection of correlations, which would increase false negatives. The network also reveals heterogeneity of 6.77, meaning some nodes have significantly more connections than others. To pinpoint which nodes may be keystone features of this network, we further investigated which individual metabolites have the highest degree centrality (most associations with other viruses, Fig. 3B). The 10 metabolites with the most associations formed 11–15 positive correlations with cell-associated viruses (Table S3). The majority of compounds are unknown, but the metabolite with the most positive links was an analog of an organonitrogen compound (X13970). This compound also had negative correlations with viruses. Two others could be identified as fatty acyls, which only formed positive associations with viruses. One fatty acyl was identified via analog hit (X34394) while the other fatty acyl (X8968) had a spectral match to 9,12-octadecadiynoic acid from the 2014 NIST Mass Spectral Library. For metabolites with the highest number of negative associations, only analogs were predicted, where hits to prenol lipids made up three of the six compounds. These compounds were unique to the negative associations. The same organonitrogen compound (X13970) that formed the highest number of positive correlations with viruses was also among the compounds that formed the highest number of negative correlations. Correlations unique to negative and positive networks can be found in Tables S4 and S5.

We also investigated the viruses with the most edges in the positively and negatively cell-associated networks (Figure 3C; Table S6). Though the difference in maximum number of links is influenced by the difference in network size, the negative network was approximately 21 times smaller, while the maximum number of links was 150 times smaller. The viruses with the highest number of positive associations formed links with 1,541 metabolites. Five of the viruses with the highest number of associations (v17VMAG56, degree centrality = 1,521; v7vMAG135, degree centrality = 1,349; v7vMAG131, degree centrality = 1,309; v7vMAG97, degree centrality = 1,278; v5vMAG114, degree centrality = 1,341) have been previously found to associate with the host *Sphingobium yanoikuyae* via proximity-ligation (23). Additionally, genome-based predictions revealed further potential host taxa for other high-ranking viruses: vRhyme_4044 was also predicted to infect a host of the genus *Sphingobium,* and three viruses were predicted to infect hosts of the class *Bacteroidia* (v5vMAG188), *Coriobacteriia* (vRhyme_4265), and *Alphaproteobacteria* (vRhyme_2005). Three hosts were predicted for negatively associated viruses: *Bacteroidia* (vRhyme_bin_3208, v5vMAG43), *Acidimicrobia* (v19vMAG90), and *Gammaproteobacteria* (vRhyme_bin_1485).

Six of the ten viruses with the highest number of positive edges were temperate (i.e., encoding an integrase or identified as a prophage), despite the positive network consisting of 90% putatively lytic viruses (acknowledging that some lytic assignments may include undetected lysogenic viruses). To further investigate differences in the links formed between putatively lytic and temperate viruses within the positive-association network, we compared the differences in Gibbs free energy from metabolites correlated with each virus (Fig. 3D). A Wilcoxon rank-sum test revealed a small but significant difference in the distribution of Gibbs energy of compounds associated with putatively lytic versus temperate viruses (Wilcoxon rank-sum test, *P*-value = 0.00404; lytic mean = 88.8 kJ [mol °C]^−1^; temperate mean = 89.9 kJ [mol °C]^−1^). This may indicate that, within the metagenome-virus-metabolite network, temperate viruses tend to associate with energy-rich compounds more so than putatively lytic viruses. To assess whether this pattern held across the core range of the data, we subsampled metabolites with Gibbs energy values between 85.1 and 101.3 kJ mol⁻¹ °C⁻¹, capturing the central mode of the distribution. Within this subset, the Wilcoxon rank-sum test did not detect a statistically significant difference between the two groups (*P* = 0.0524). However, a Kolmogorov–Smirnov test revealed a significant difference in the overall shape of the distributions (*D* = 0.061442, *P* = 0.001347), indicating that the frequency and/or spread of metabolite associations differ between putatively temperate and lytic viruses, with a potential enrichment of temperate virus associations in specific energy ranges.

### Correlations between virome-derived viral abundances and metabolites

**Fig 4:**
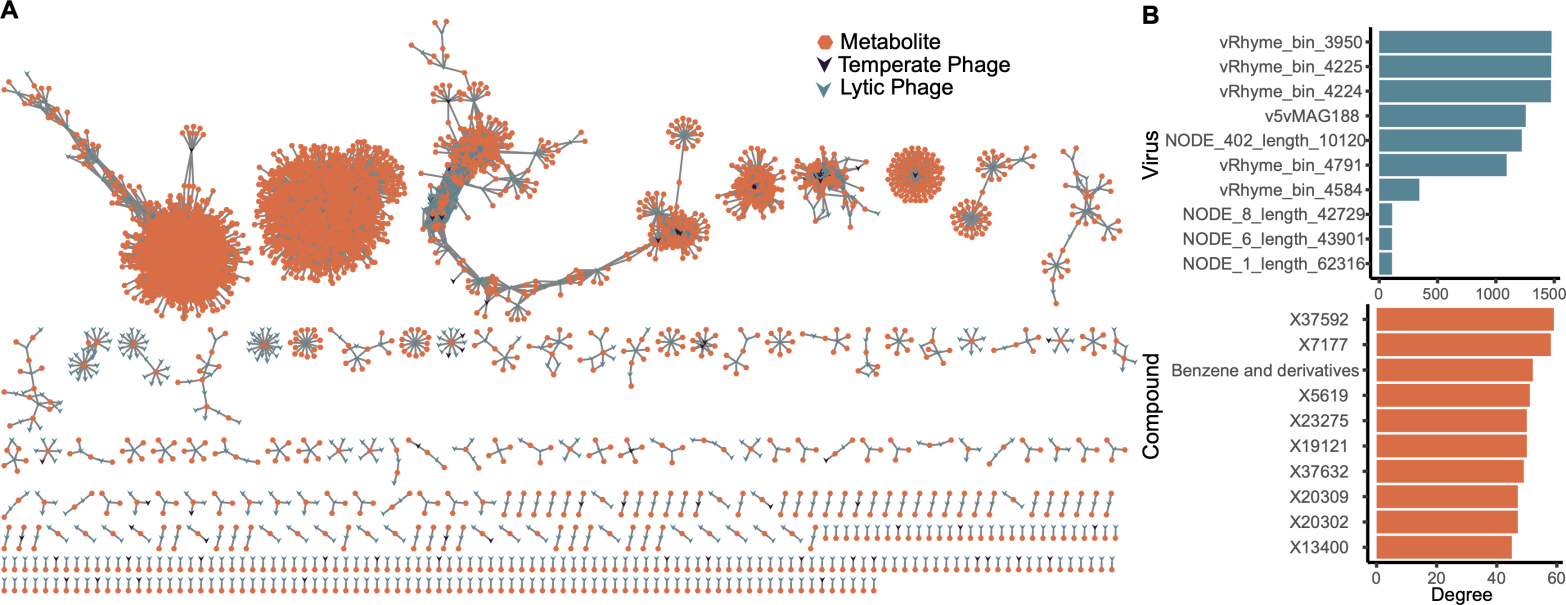
Network of positive associations between free viral abundances and metabolites. (**A**) Network of positive associations between phages (<0.45 mm fraction, virome) and metabolites. V-shaped nodes represent phages and hexagon-shaped nodes represent metabolites. Edges represent significant positive correlations (Benjamini-Hochman corrected Pearson’s, *P* < 0.01). (**B**) Viruses with the most connections with positive associations (top) and metabolites with the most connections with positive associations (bottom).

A second network was built with viruses from the free virus fraction (virome, <0.45 µm) and metabolites. The positive virome network had 4,972 nodes, of which 4,191 were metabolites, and 781 were viruses, forming 14,466 positive associations (Fig. 4A), about 1.25 times smaller than the metagenome-derived (>0.22 µm) network. Similarly, the negatively associated virome network was smaller than the positive network, with 1,059 total nodes and 733 links (Fig. S7). This network also showed a heterogeneity of 8.71, indicating that some nodes formed more associations than others. The viruses with the highest number of associations correlated with 110 to 1,477 metabolites, while the metabolites with the highest number of associations correlated with 45–59 viruses (Fig. 4B). Three of these viruses encoded predicted metabolic genes: vRhyme_bin_3950, v5vMAG188, and NODE_1_length_62316_cov_79.381459, which were all related to pyrimidine metabolism (deoxyribonucleotide biosynthesis). Predicted hosts belonged to the class *Bacterioidia* (vRhyme_bin_4225 and v5vMAG188), *Bacilli* (vRhyme_bin_4224), and *Gammaproteobacteria* (vRhyme_bin_4584). Of the viruses with the highest number of negative associations, four were predicted to infect bacteria of the class *Bacteroidia,* and two were predicted to infect *Alphaproteobacteria* (Fig. S8a; Table S6). The metabolic compounds were predominantly unknown, except for one belonging to the analog class benzene and derivatives, which was only found in the positive correlation network (Fig. 4B). This compound had a spectral match with fexofenadine (Massbank: EX301902). The metabolites with the most negative associations (Fig. S8b; Table S6) had an analog hit to steroids and derivatives (X11307), and two known compounds, diethylene glycol monobutyl (X332, GNPS Spectrum ID: CCMSLIB00005726830) and DEET (X1847, GNPS Spectrum ID: CCMSLIB00000579756), which were only identified in the negative correlation networks (Table S5). This network also differed from the metagenome-derived network, where there were no temperate viruses with a high number of linkages or a significant difference in the Gibbs free energy of compounds correlated with putatively temperate or lytic viruses (Wilcoxon rank-sum test, *W* = 869,807, *P*-value = 0.857).

### Differences between compounds associated with putatively temperate and lytic viruses

**Fig 5:**
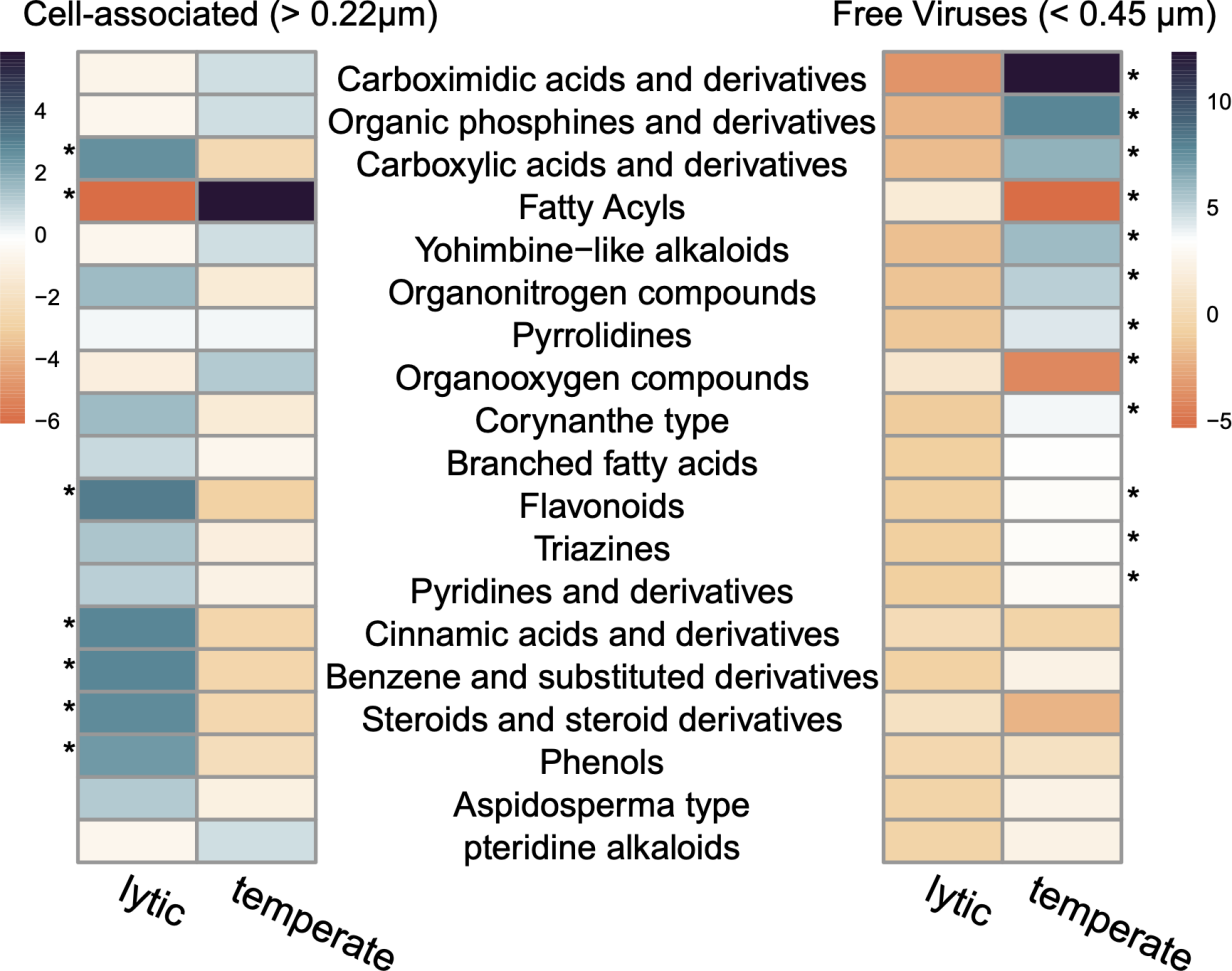
Associations between metabolites and viral replication strategy. The heatmap displays Pearson’s residuals >2 for the positive metagenome virus-metabolite network (left) and the positive virome virus-metabolite network (right). According to a global Fisher’s exact test with Monte Carlo simulation (50,000 iterations), both networks exhibit significant differences in compound associations (*P* < 0.0001 for the metagenome network and the virome network). Asterisks denote significant (*P* < 0.05) pairwise Fisher’s exact tests with FDR correction for multiple comparisons.

To further investigate differences between compounds associated with putatively lytic and temperate viruses, we performed a global Fisher’s exact test with Monte Carlo simulations (50,000 repetitions). The analysis revealed a significant association between viral lifestyle (lytic vs. temperate) and the compounds they interact with in both the positive cell-associated-virus-metabolite network (*P*-value < 0.0001) and the free-viruses-metabolite network (*P*-value < 0.0001). To identify the specific groups of compounds driving these differences, we conducted pairwise Fisher’s exact tests with FDR correction, revealing seven compounds driving significant differences (*P*-value < 0.05) with cell-associated viruses (fatty acyls, cinnamic acids and derivatives, flavonoids, benzene and substituted derivatives, steroid and steroid derivatives, carboxylic acids and derivatives, and phenols; Table S7) and 12 compounds associated with free viruses (carboximidic acids and derivatives, fatty acyls, carboxylic acids and derivatives, organic phosphines and derivatives, organooxygen compounds, organonitrogen compounds, yohimbine-like alkaloids, pyrrolidines, corynanthane type, flavonoids, pyridines and derivatives, triazines; Fig. 5; Table S8). Pearson residuals, which indicate variables contributing disproportionately to the observed association, agree with significant correlations between compounds and lifestyle (Tables S9 and S10).

In the positive cell-associated-metabolome network, fatty acyls exhibited the strongest deviation from independence between viral lifestyles (Fishers’ exact test, *P*-value = 2.3 × 10^−23^). This pattern reflects a significantly greater-than-expected number of fatty acyl associations with temperate viruses and a corresponding depletion in lytic viruses, as indicated by the Pearson residuals (temperate = 5.86, lytic = –6.13; Fig. 5).

The pattern observed for fatty acyls in the cell-associated network was reversed in the free-virus network, where fatty acyls significantly contributed to differences in viral lifestyle (*P* = 1.38 × 10^−15^) but lytic viruses were enriched, as shown by Pearson’s residuals (temperate = –5.40, lytic = 1.53). In the positive free-virus network, carboximidic acids and derivatives were the most strongly associated with temperate viruses (*P*-value = 9.86 × 10^−15^, Pearson residuals lytic = −3.49, temperate = 12.26). By comparing residuals between the two networks, we found that all shared significant compound classes exhibited inverted associations. Specifically, carboxylic acids and flavonoids were significantly associated with putatively lytic viruses in the metagenome network but with temperate viruses in the virome network. On the other hand, fatty acyls were significantly associated with temperate lifestyles in the cell-associated fraction but with lytic lifestyles in the free virus network.

## DISCUSSION

Here, we show a significant relationship between the composition of the DOM pool and viral replication strategies on coral reefs. Cell-associated phages showed more frequent correlations with compounds of lower NOSC values (Fig. 2B), and temperate phages, in particular, were associated with more reduced, energy-rich metabolites (Fig. 3C). Although the directionality of these correlations cannot be determined, this observation supports the notion that lysogeny may be selected for an environment with an abundant supply of high-energy molecules. This is consistent with observations of low virus-to-microbe ratios on microbialized reefs with a supply of highly labile, algae-derived DOM (14, 19, 50, 66). In the following sections, we discuss two alternative hypotheses to explain the potential directionality of these relationships: (i) the potential effects viruses may have on exometabolome profiles via bacterial cell lysis and (ii) the potential effects that coral- and algae-derived metabolites could have on the viral community composition and replication strategies. We follow these discussions with a note on limitations and future directions.

### Top-down control and functional modulation: potential for viruses affecting the reef exometabolome composition

Phages may influence DOM composition through bacterial lysis (67, 68). However, tropical marine bacteria consist of approximately 12.4 fg of carbon per cell (69), and with cell densities of roughly 10^6^ cells per mL, contribute to about 1.03 µM of DOC. Considering that reefs in this study displayed on average 86.5 µM of DOC (SD = 5.7), lysis of the entire bacterial community would still only make up 1.19% of the total DOC fraction. Although this contribution may be small, these compounds may disproportionately affect microbial community structure (70, 71). However, because coral reefs’ largest fractions of DOM are derived from macroalgae exudates and coral mucus (8, 12, 72) and filtering methods bias against low-abundance compounds, our study is likely unable to detect subtle changes in DOM due to bacterial lysis.

Alternatively, viruses may influence DOM composition by modulating bacterial physiology, such as by enhancing or decreasing the efficiency of hosts metabolizing substrates, thereby shifting their NOSC values. This modulation could result from the expression of phage-encoded metabolic genes that augment host metabolism (26, 73–77). For instance, cyanophages can carry photosynthesis genes *psbA* and *psbD* to sustain energy production during infection, ensuring sufficient ATP generation for viral replication (78). Because bacteria are estimated to consume around 1.7%–28.5% of DOC released by benthic primary producers (12, 79), it is possible that changes induced through viral infection or transduction may affect the nutrient uptake of bacteria and hence affect the DOM composition left behind. However, further experiments are required to test the prominence of these changes in bacterial consumption and whether they scale up to affect the composition of the total DOM pool.

### Evidence for compounds affecting viral replication strategy

An alternative scenario for the relationship between phages and metabolites is that the presence of energy-rich and highly reduced compounds may selectively favor specific bacterial taxa, which, in turn, affects the viral community. Low-NOSC compounds require higher oxidative power for degradation (80), and when energetic supply exceeds oxidative capacity, net ATP yield can decrease, leading to dissipation pathways and overflow metabolism (81, 82). The concept of increased electron donor to acceptor ratio (e^−^DAR) on coral reefs has been hypothesized to create conditions favorable for lysogeny (8, 12, 66). A higher e^−^DAR would result in ATP depletion coupled to an excess of NADPH, which promotes the accumulation of putatively lytic phage repressors in model phage and bacteria (66). This may facilitate both new lysogenic infections and the maintenance of existing prophages (83). Such a mechanism may explain the relationship between temperate viruses and more energy-rich compounds (Fig. 3D).

This mechanism is also consistent with the observation that the six most interconnected viruses within the metagenome-associated network were temperate (Fig. 3C), despite the vast majority (>90%) of the network consisting of putatively lytic viruses. Notably, five of these viruses shared the same predicted host species, *Sphingobium yanoikuyae*, a heterotrophic bacterium frequently found in coral reef environments (25, 84, 85). *Sphingobium* species are known for their ability to degrade environmental pollutants, including complex hydrocarbons, making them key candidates for bioremediation (86–88). Aromatic degradation requires substantial molecular oxygen and energy input (89) and may lead to ATP depletion and oxygen limitation, potentially creating favorable conditions for phage integration.

Fisher tests revealed a significant association between viral lifestyle and compound class across both networks, with the compounds contributing most strongly to this pattern exhibiting opposite associations with putatively lytic and temperate viruses in the metagenome- versus virome-derived data sets (Fig. 5). This may further reflect evidence for compounds affecting viral replication strategies. A temperate virus must undergo lysis to be abundant in seawater, whereas a higher abundance in the cell-associated fraction may be due to lysogenic or dormant infection. Therefore, a highly abundant temperate phage in seawater likely indicates that a phage is undergoing a lytic cycle, while its prevalence in the cell-associated fraction points to lysogeny. The stronger relationship observed between energy-rich compounds and cell-associated viral abundances (Fig. 2B), but not free viruses (Fig. 2C), may reflect that metagenome-derived viruses are more tightly linked to host abundances (26), including integrated prophages. This distinction helps explain the observed inverse relationship between compounds in these two fractions. Therefore, both independent networks highlight similar trends between putatively lytic and lysogenic cycles.

We note that the virus-metabolite correlations discussed here should be interpreted with caution, given the small sample size, which could lead to false-positive signals and the possibility that viruses classified as lytic may be overrepresented. This overestimation can occur when integration genes are not detected, leading to potential misclassification of a temperate virus as lytic. Future studies with a larger sample size covering a wider range of NOSC values and microbial densities across larger geographical scales are required to test the consistency of these relationships, and manipulative experiments are required to test the causal inferences.

### Implications for coral reef biogeochemistry and functioning

Our findings are conceptually consistent with the DDAM framework and prior work on microbialization, but should be interpreted with important caveats. Corals in Curaçao have decreased in cover from 32.6% to 9.2% between 1973 and 2013, with fleshy algae and cyanobacteria mats increasing in cover (90). Although we did not identify the sources of individual compounds, there is evidence that both labile, energy-rich algal exudates (12, 15, 16) and high molecular weight coral exudates (14) lead to enrichments of opportunistic heterotrophic microbial communities. The strong associations with fatty acyls and the predominant presence of arabinose and glucosamine among carbohydrates indicate that the metabolomes here resemble coral exudates more than algae (14). Stress-induced mucus production and sloughing in corals may contribute energy-rich organic matter to the surrounding environment (91), which would increase during DDAM dynamics. Perturbations to ambient DOC through high-energy compounds have been shown to alter microbial communities and hypothesized to increase lysogeny through the e^−^DAR mechanism described above (81). We observed associations between high-energy DOM and viral signals consistent with lysogeny (Fig. 2B, 3C and D). Compounds with high Gibbs energy values have also been associated with the enhanced growth of opportunistic coral pathogens (16), further reinforcing the potential for coral disease. Together, these indirect patterns may reinforce DDAM feedback by allowing the persistence of virulent microbial communities through lysogeny. These proposed causal mechanisms and ecological outcomes should be the focus of future studies.

## Supplementary Material

Reviewer comments

## Data Availability

Raw sequence reads are available through the NCBI Sequence Read Archive (SRA) under PRJNA975592. Virus and exametabolome data are available through Figshare as follows: fasta file with viral genome sequences (vMAGs and contigs), https://doi.org/10.6084/m9.figshare.23313773.v1; viral sequence unique ID, length, and abundance, https://doi.org/10.6084/m9.figshare.28924508.v2; exometabolite unique ID and abundance, https://doi.org/10.6084/m9.figshare.28924043.v2. Bash scripts used to process sequence data are available via GitHub (https://github.com/Silveira-Lab/Varona-et-al-2023-Phage-host-network-structures).

## SUPPLEMENTAL MATERIAL

Supplemental figures msystems.00395-26-s0001.pdf Fig. S1–S8.

Supplemental Tables msystems.00395-26-s0002.xlsx Tables S1–S10.

OPEN PEER REVIEW reviewer-comments.pdf An accounting of the reviewer comments and feedback.

ASM does not own the copyrights to Supplemental Material that may be linked to, or accessed through, an article. The authors have granted ASM a non-exclusive, world-wide license to publish the Supplemental Material files. Please contact the corresponding author directly for reuse.
