# Supplementary material for: Bacteriophage replication strategies are associated with organic matter energy content on coral reefs
Source: mSystems. 2026 Jun 22;11(7):e00395-26. doi: 10.1128/msystems.00395-26 (PMC13386980; doi:10.1128/msystems.00395-26)
Supplement: Supplemental figures — Fig. S1–S8. [file msystems.00395-26-s0001.pdf]

Supplementary Figures

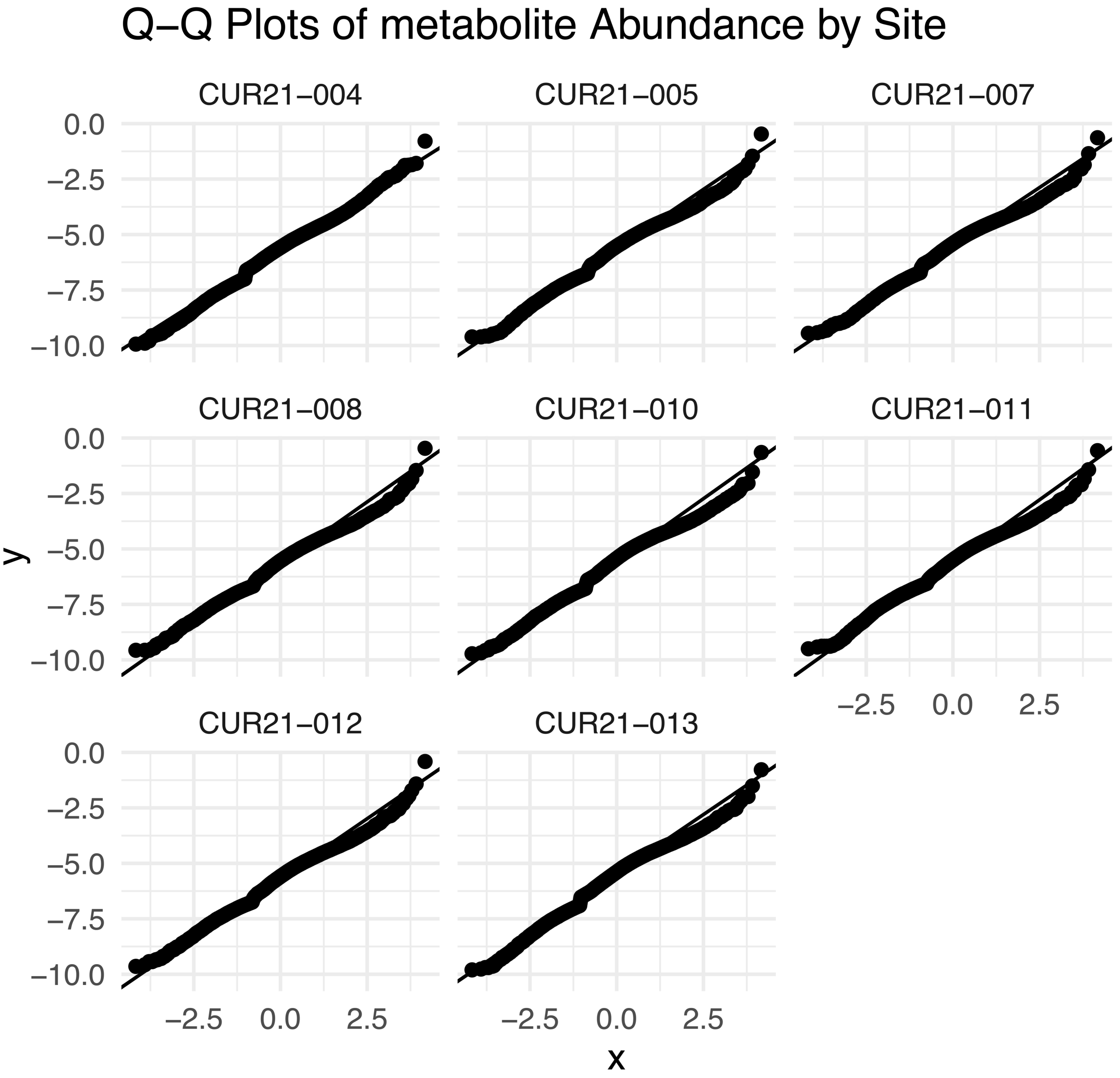

Figure S1: Q-Q plots of metabolite abundances used for network generation.

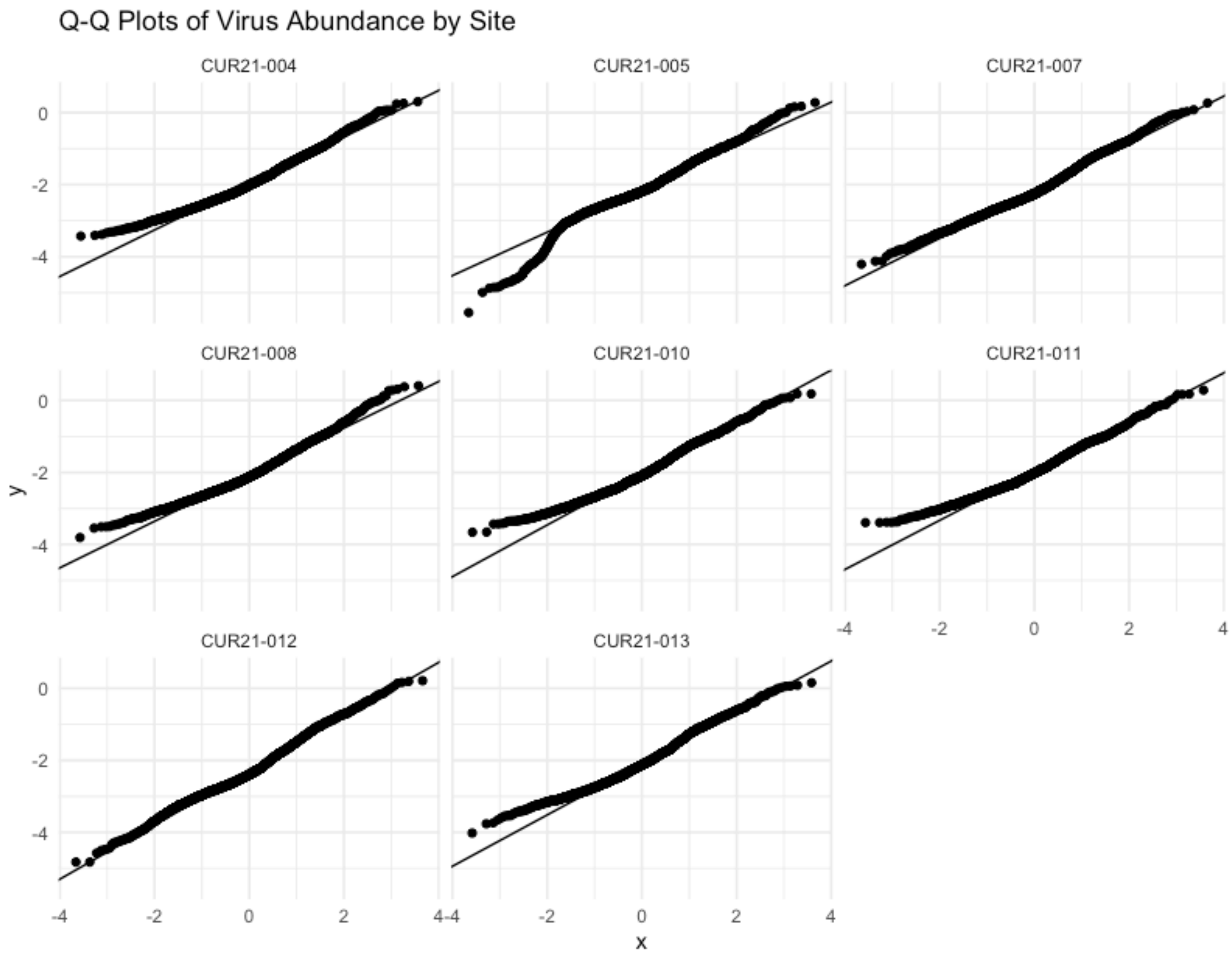

Figure S2: Q-Q plots of virus abundances from metagenome fraction (>0.22 µm) used for network generation.

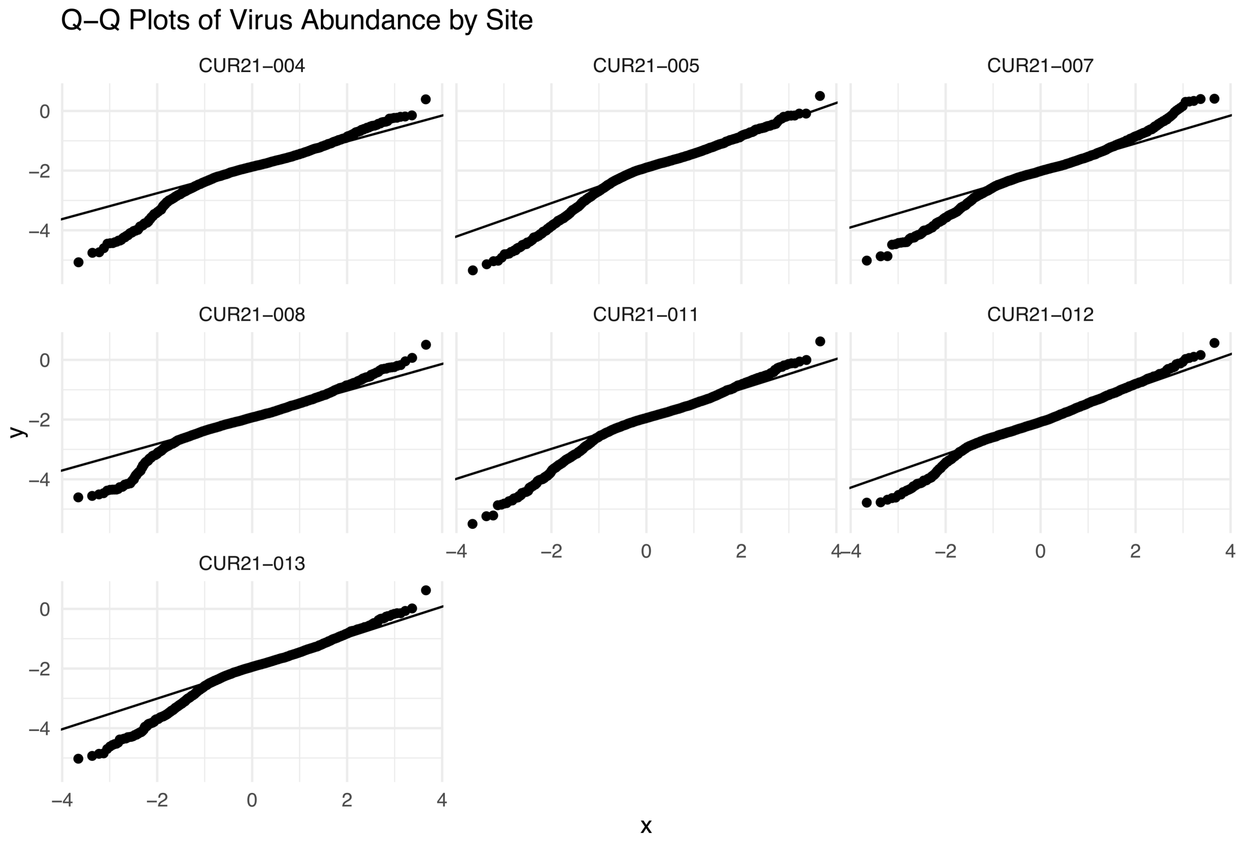

Figure S3: Q-Q plots of virus abundances from virome fraction (<0.45 µm) used for network generation.

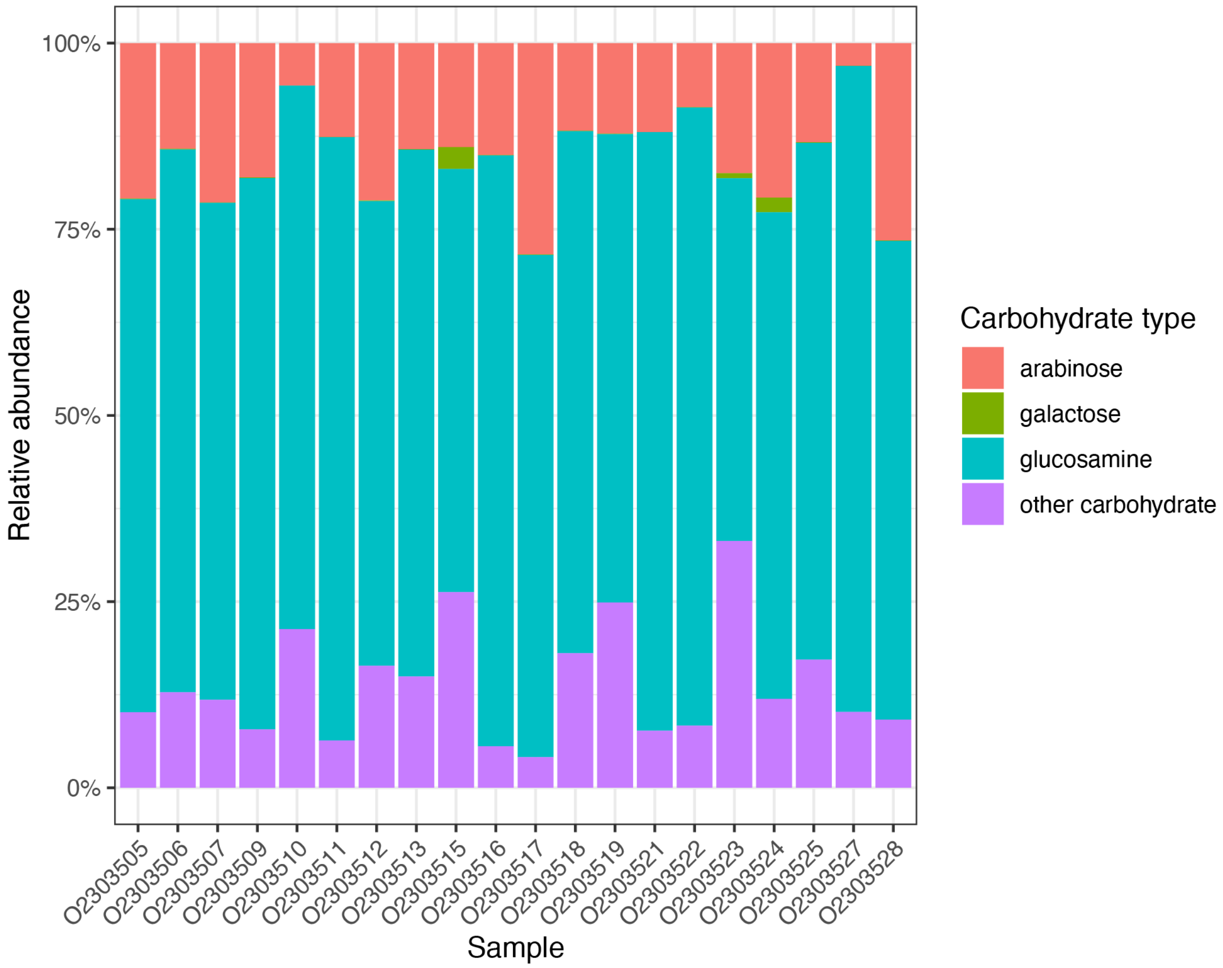

Figure S4. Relative abundances of classified sugars. Carbohydrates were identified using search terms "carbohydrate", "sugar", "galactose", "fucose", "xylose", "mannose", "arabinose", and "glucosamine”.

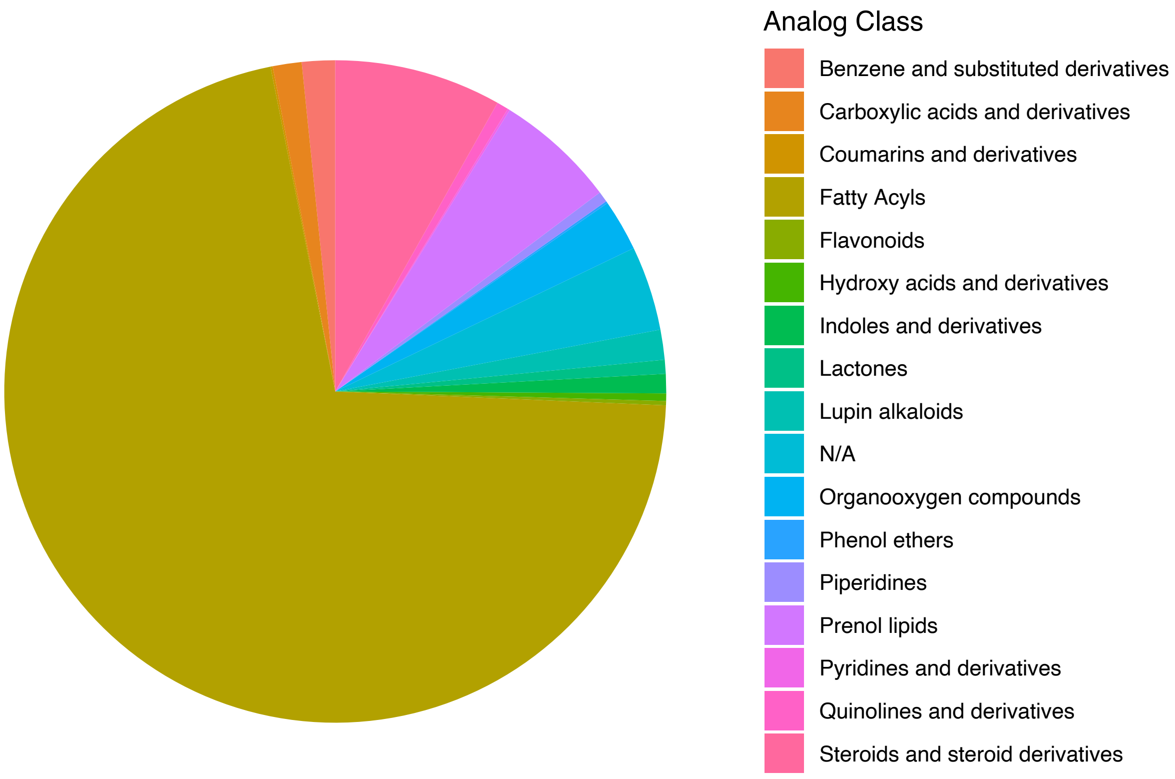

Figure S5. Distribution of chemical classes of exometabolome analogs ranging from NOSC values -1.45 to -1.18. These compounds are from first peak in Fig. 2b. The majority of these compounds were made up of fatty acyls (71.1%).

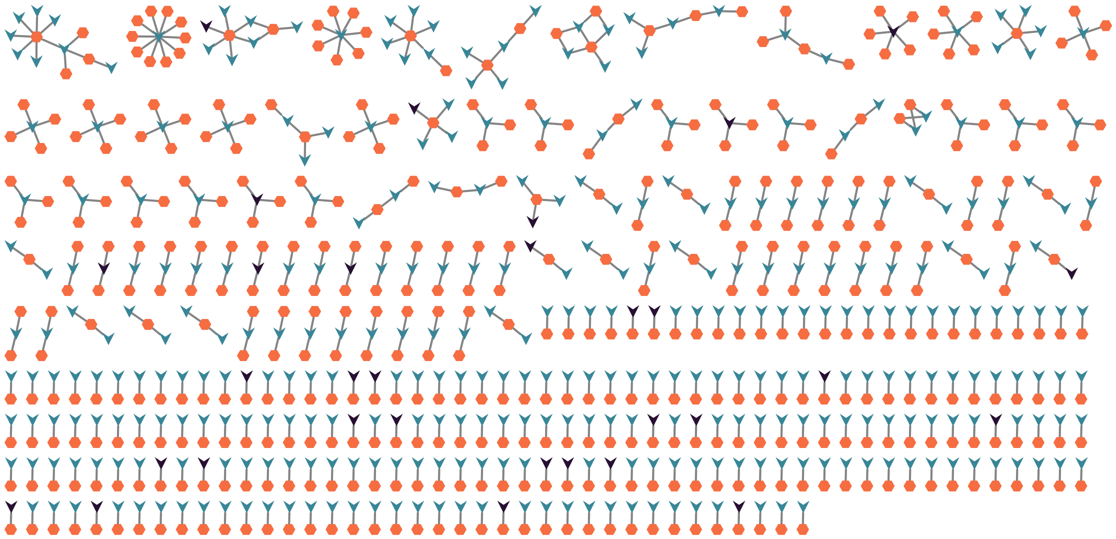

Figure S6. Negative association between cell-associated virus-metabolite network. The network shows 831 connections between metabolites which are displayed as orange hexagons, and the lytic viruses as teal "V"shaped and temperate viruses as dark blue "V"-shaped.

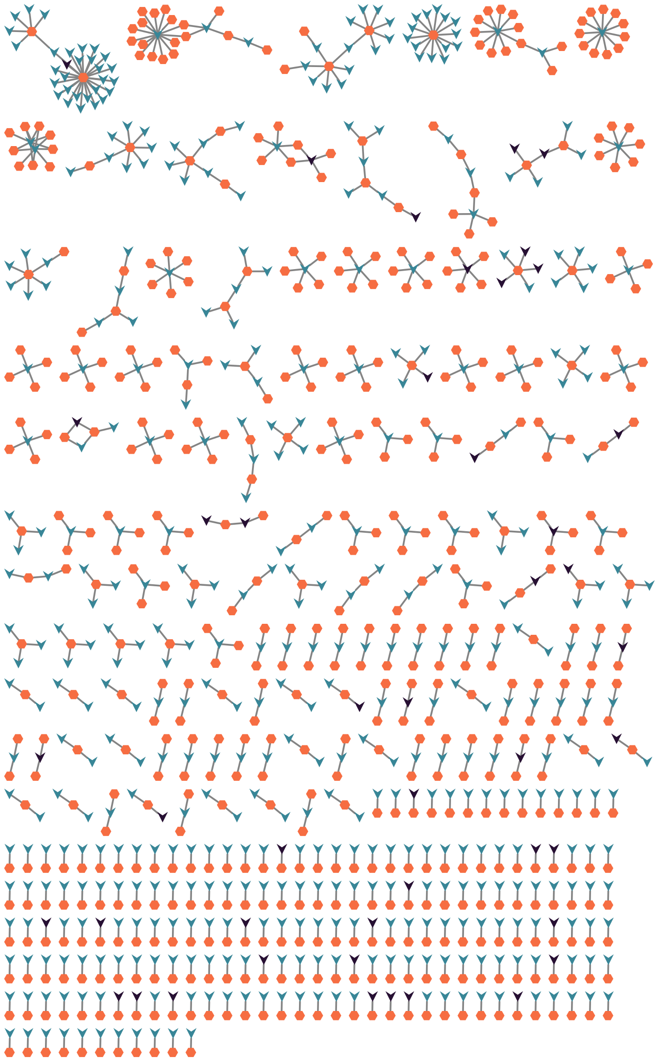

Figure S7. Free-virus-metabolome negative associations. There are a total of 1059 total nodes and 733 links between metabolites which are displayed as orange hexagons, and the lytic viruses as teal "V-shaped and temperate viruses as dark blue "V"-shaped.

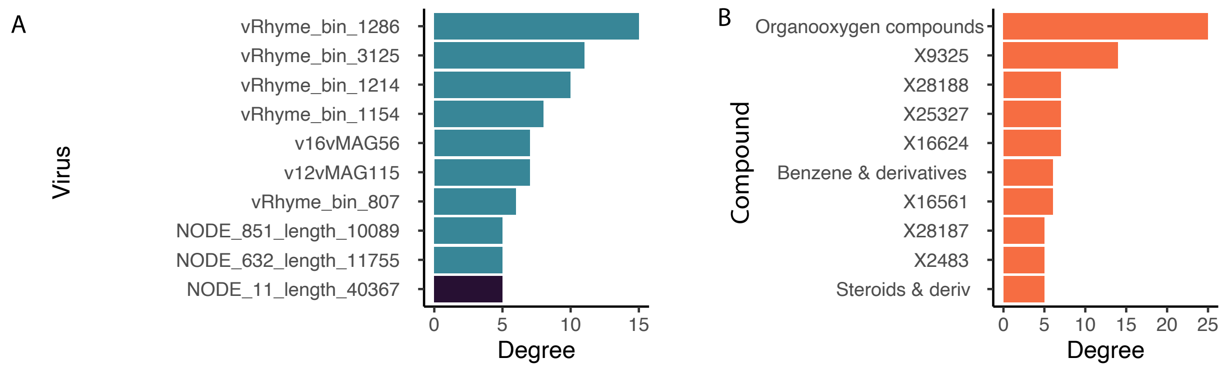

Figure S8. Viruses and compounds with the most negative associations from free-virus-metabolite association network. (A) Viruses with the most negative associations with metabolites (B) metabolites with the most negative associations.
